# Zinc transporters in Alzheimer’s disease

**DOI:** 10.1186/s13041-019-0528-2

**Published:** 2019-12-09

**Authors:** Yingshuo Xu, Guiran Xiao, Li Liu, Minglin Lang

**Affiliations:** 1 CAS Center for Excellence in Biotic Interactions, College of Life Science, University of Chinese Academy of Sciences, Beijing, 100049 China; 2 School of Food and Biological Engineering, Hefei University of Technology, Hefei, 230009 China; 3 State Key Laboratory of Brain and Cognitive Science, Institute of Biophysics, Chinese Academy of Sciences, Beijing, 100101 China; 4 College of Life Science, Hebei Agricultural University, Baoding, 071001 China

**Keywords:** Zinc transporter, Brain, Genetic modulation, Emerging therapeutic target, Alzheimer’s disease, Ageing

## Abstract

Alzheimer’s disease (AD) is the most devastating neurodegenerative disorder. Due to the increase in population and longevity, incidence will triple by the middle of the twenty-first century. So far, no treatment has prevented or reversed the disease. More than 20 years of multidisciplinary studies have shown that brain zinc dyshomeostasis may play a critical role in AD progression, which provides encouraging clues for metal-targeted therapies in the treatment of AD. Unfortunately, the pilot clinical application of zinc chelator and/or ionophore strategy, such as the use of quinoline-based compounds, namely clioquinol and PBT2, has not yet been successful. The emerging findings revealed a list of key zinc transporters whose mRNA or protein levels were abnormally altered at different stages of AD brains. Furthermore, specifically modulating the expression of some of the zinc transporters in the central nervous system through genetic methods slowed down or prevented AD progression in animal models, resulting in significantly improved cognitive performance, movement, and prolonged lifespan. Although the underlying molecular mechanisms are not yet fully understood, it shed new light on the treatment or prevention of the disease. This review considers recent advances regarding AD, zinc and zinc transporters, recapitulating their relationships in extending our current understanding of the disease amelioration effects of zinc transport proteins as potential therapeutic targets to cure AD, and it may also provide new insights to identify novel therapeutic strategies for ageing and other neurodegenerative diseases, such as Huntington’s and Parkinson’s disease.

## Introduction

Alzheimer’s disease (AD) is a progressive neurodegenerative disease, which leaves many elderly people cognitively impaired [1] and the numbers are expected to reach 131 million by 2050 [2]. The disorder is characterized by a progressive loss of memory due to the degeneration and/or loss of neurons related to cognition in the hippocampus and cortex. Pathologically, the extracellular accumulation of amyloid plaques rich in the β-amyloid (Aβ) peptide and intracellular deposition of neurofibrillary tangles (NFTs) with hyperphosphorylated tau in the brain are the two major hallmarks of AD [3, 4]. Aβ aggregates can form complexes with redox active metals, and the phosphorylation of tau is also associated with metals [5], especially zinc, which interplays with the Aβ protein, aggravating the precipitation of senile plaques, and it can also bind to tau proteins, contributing to the formation of NFTs [6–8]. Considerable early clinical studies, including pharmaceutical approaches designed to change the level of zinc or to alter the distribution of zinc and copper, have provided encouraging clues for metal-targeted therapies in the treatment and prevention of AD [9–11].

Zinc metabolism is firmly controlled by zinc transporters that transport zinc through the membrane to the cytoplasm or organelles and maintain zinc homeostasis both at the cellular and systemic levels. Zinc transporters are classified into two families: the ZnT (SLC30) family and the ZIP (SLC39) family. To the best of our knowledge, most zinc transporters are also expressed in mammalian brains, such as ZnT1, ZnT3, ZnT10, ZIP1 and ZIP6 [12–17]. Moreover, it has been established that a considerable number of zinc transporters were abnormally expressed in the brains of AD patients and APP/PS1 mice [18–20], suggesting that zinc transporters may play a critical role in the progression of AD. Since so far there has been no effective therapy to halt or reverse AD, we propose that altering zinc homeostasis by modulating the expression or capability of zinc transporters may be a better way to ameliorate AD. This review focuses first on the relationship between zinc transporters and AD. After that, we summarize the function of zinc transporters that are involved in brain zinc homeostasis and assess recent evidence for treating AD with the alteration of zinc transporters.

## The important role of zinc in the brain

As an indispensable trace element, zinc is a structural or functional component of many proteins. It also participates in various physiological functions, including but not limited to DNA synthesis, brain development and neurotransmission [21–23]. Considering its multifaceted role in biological processes, alterations in zinc concentrations from normal levels can induce many devastating diseases, e.g., zinc deficiency leading to mental lethargy, growth retardation and immune dysfunction, while zinc overload can affect the function of lymphocytes and cause copper deficiency [24].

Besides the important role of zinc in normal mammalian brain development and physiology [25], it also plays a critical role in the function of the central nervous system (CNS). There are three pools of zinc in the brain, namely vesicular zinc, protein-bound zinc and free zinc [26]. Vesicular zinc exists mainly in glutamatergic neurons, but not all glutamatergic neurons contain zinc [27, 28]. Studies found that zinc can permeate through the neuronal membrane via several ion channels, such as Ca^2+^-permeable α-amino-3-hydroxyl-5-methyl-4-isoxazolepropionate-acid (AMPA) /kainite (Ca^2+^-A/K) channels [29], high-voltage activated (HVA) Ca^2+^ channels [30], and N-methyl-D-aspartate (NMDA) receptor-gated channels [31], depending on neuronal stimulation. Zinc binds to the extracellular amino-terminal domain (ATD) of NMDA receptors [32], and activation of AMPA and NMDA receptors facilitated Zn^2+^ influx into neurons [31, 33]. Zinc can also enter neurons through voltage-gated calcium channels, especially when the pH is lower [30]. Toxic activation studies have shown that Ca^2+^-A/K channels can carry a greater flux of zinc than influx of zinc in comparison with voltage-sensitive Ca^2+^ channel (VSCCs) and NMDA channels, and the permeability of Ca^2+^-A/K channels to zinc is also comparable to their permeability for Ca^2+^ [29]. Because they are concentrated in postsynaptic regions of dendrites adjacent to sites of presynaptic release, the Ca-A/K channels may constitute the principal physiologic route for Zn^2+^ translocation [29]. Zinc functions as a regulator of many enzymes and can directly interact with mitochondria and cause reactive oxygen species (ROS) production leading to important physio-pathological consequences, including neurodegeneration [29, 34]. Moreover, there is increasing evidence that high level of intracellular free zinc can induce neuronal death by inhibiting the process of energy production through interacting with targets essential to mitochondrial electron transport, the tricarboxylic acid cycle (TCA), and glycolysis [35], and the consequences of zinc overload also include reduced cellular ATP levels and loss of mitochondrial membrane potential [35]. A later study through direct biochemical assays demonstrated that excess zinc can irreversibly damage the major enzymes of energy production and antioxidant defense prior to mitochondrial permeability transition [36]. Zinc will participate in numerous signal transduction processes as soon as it enters the postsynaptic neuron, and as a neuromodulator, zinc can influence the brain excitability by inhibiting excitatory and inhibitory receptors, thus Zn^2+^ qualifying as both a neurotransmitter and a second messenger [37] .

On the other hand, zinc can modulate synaptic plasticity in the form of long-term potentiation (LTP) and long-term depression (LTD), respectively, which are considered to be underlying learning and memory, and are differentially modulated by NMDA receptor subunits [37–39]. When stimulated, zinc is co-released with glutamate into the synaptic cleft to modulate the activity of post-synaptic proteins, NMDA and AMPA receptors. Simultaneously, zinc can transactivate the tropomyosin-related kinase B (TrkB) receptor in cultured cortical neurons. As soon as zinc enters the postsynaptic neuron, it activates brain-derived neurotrophic factor (BDNF) signaling in a neurotrophin-independent manner [40, 41]. Zinc can also affect BDNF signaling by promoting maturation of pro-BDNF to BDNF all through the activation of matrix metalloproteinases (MMPs), thereby significantly increasing BDNF level in brains of AD mice and greatly delaying hippocampal-dependent memory deficits [42]. A subsequent study has shown that under physiological conditions, it is BDNF rather than vesicular zinc that activates TrkB in hippocampal mossy fiber axons [43]. Synaptic release of Zn^2+^ activates a Zn^2+^-sensing receptor, mZnR/GPR39, induces Ca^2+^-signaling, then activates ERK1/2 MAPK and up-regulates clusterin; however, Zn^2+^ signaling via mZnR/GPR39 is disrupted by amyloid-β in AD brains, which is a critical pathological component of AD [44]. Such a vital role of zinc in the brain has prompted the scientific community to continue exploring the etiology and therapeutic strategies based on zinc dyshomeostasis in neurodegenerative diseases, especially for AD, Parkinson’s disease (PD) and Huntington’s disease (HD) [1, 37, 45].

## The maintenance of zinc homeostasis in human and mammalian cells

**Fig. 1:**
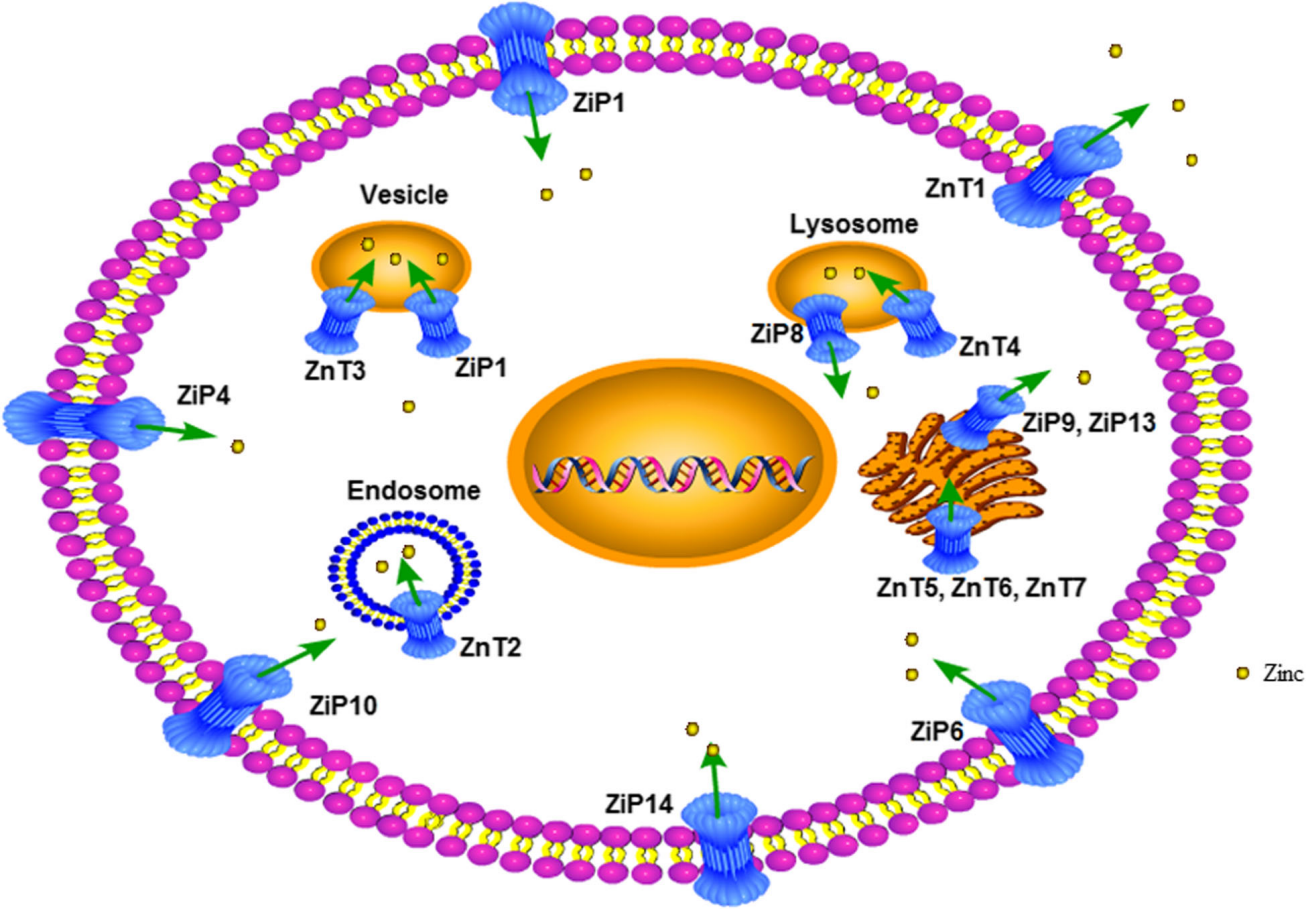
An overview of the locations of some crucial zinc transporters in mammalian cells and the intracellular and intercellular compartmentation of zinc ions. Zinc transporters are classified into two major families, the ZnT (SLC30) family and the ZIP (SLC39) family. As indicated by arrows in the figure, the general functions of ZnTs (ZnT1–7) is to reduce cytoplasmic zinc levels through mobilization of zinc out of cells or into intracellular compartments from the cytoplasm, whereas the function of ZiPs (ZiP1, ZiP4, ZiP6, ZiP8–10, ZiP13–14) is to increase cytoplasmic zinc levels through absorbing zinc into cytoplasm from extracellular space or mobilizing zinc out of intracellular compartments

Endogenous zinc equilibrium is promoted by zinc-binding proteins (metallothioneins, MTs) and zinc transporters. There are four MTs in human and mammalian cells: MT-1, MT-2, MT-3 and MT-4, which can bind up to seven divalent metals, including Zn^2+^, Cu^2+^, Mn^2+^, Fe^2+^ and Cd^2+^, etc. MT-1 and MT-2 are widely expressed, MT-3 is abundant in the brain, and MT-4 is expressed in some epithelial cells. When zinc is in excess, MTs may function in sequestration of excess zinc, thereby protecting cells from zinc toxicity [46]. Meanwhile, zinc transporters are responsible for the movement of zinc across the membrane; they either pump zinc out of the cytoplasm or load zinc into the intracellular space. Generally, zinc transporters are divided into two major families: the SLC30 family (ZnTs1–10) and the SLC39 family (ZIPs1–14) (see Fig. 1). The SLC30 family of zinc transporters liberates zinc out of the cytoplasm or into cellular organelles, resulting in the reduction of zinc concentration in the cytoplasm. In contrast, the SLC39 family of zinc transporters increase intracellular zinc level by moving zinc into the cytoplasm, either from the organelles or the extracellular space [14, 47].

Most ZnT proteins have six transmembrane domains (TMDs), with a long His-rich loop between TMDs Ις and ς, which may function as a sensor of cytosolic zinc levels. The C- and N- termini of ZnTs are located inside the cytoplasm [14, 47]. ZnT proteins usually form homodimers, except ZnT5 and ZnT6, which form heterodimers to transport zinc to the secretory pathway, where zinc is loaded into the zinc-requiring enzymes [48].

ZIP proteins are distinct from ZnTs in many aspects. Most ZIP proteins have eight TMDs with a His-rich domain between TMDs ΙΙΙ and Ις, and hydrophilic residues in TMD ς that is thought to function as a metal sensor. The C-and N-termini of ZIP proteins faces the extracytoplasmic space [14, 47].

There is a growing body of evidence showing that ZnTs and ZIPs play a fundamental role in maintaining zinc homeostasis, which profoundly affects human health, either directly or indirectly. Mutations in ZnTs and ZIPs have been found to be closely associated with a considerable number of human diseases, such as ZIP4 in Acrodermatitis enteropathica (AE), ZIP13 in the spondylocheiro dysplastic form of Ehlers–Danlos syndrome (SCD-EDS), and ZnT10 in Parkinson’s Disease [47, 49]. Consequently, studies on ZnTs and ZIPs are receiving an increasing clinical interest.

## Zinc transporters in the brain

The mechanism of zinc homeostasis in the brain is complex. Elevated or reduced serum zinc has little effect on the zinc content in the brain. This indicates that zinc homeostasis in the brain is rigorously controlled and is not easily altered by peripheral zinc. The primary group of proteins involved in maintaining zinc homeostasis in an elaborate manner are zinc transporters, and most of them demonstrate an age-dependent alteration in the brain [50–52]. Table 1 lists the expression of zinc transporters detected in human or mouse brains and their alterations in the progression of AD, including at least seven ZnT (ZnT1, ZnT3, ZnT4, ZnT5, ZnT6, ZnT7 and ZnT10) and six ZIP (ZIP1, ZIP3, ZIP4, ZIP6, ZIP9 and ZIP13) transporters.

**Table 1:** Summary of brain zinc transporters and their alterations in the progression of AD

Gene name	Alias	Tissue/ Subcellular localization	Neurological disease	Expression levels in the progression of AD
*SLC30A1*	*ZnT1*	Dendritic spines, synaptic membranes [12]	Alzheimer’s Disease [53]	Decreased in PCAD, increased in both EAD and LAD [20, 53]
*SLC30A3*	*ZnT3*	Hippocampus, cortex [54]	Alzheimer’s Disease [19], Learning deficit and memory loss [18]	Decreased [19]
*SLC30A4*	*ZnT4*	Endosomal/secretory vesicles [53]	Alzheimer’s Disease [55]	Increased [55]
*SLC30A5*	*ZnT5*	Golgi apparatus [48]	Alzheimer’s Disease [56]	Unknown
*SLC30A6*	*ZnT6*	Golgi apparatus [48]	Alzheimer’s Disease [53, 57]	Increased [53, 55]
*SLC30A7*	*ZnT7*	Golgi apparatus [58]	Alzheimer’s Disease [56]	Increased [59]
*SLC30A10*	*ZnT10*	Brain and liver [14]	Parkinson’s Disease [49] Alzheimer’s Disease [60]	Decreased [60]
*SLC39A1*	*ZIP1*	Hippocampus, thalamus [15]	Alzheimer’s Disease [61]	Increased [61]
*SLC39A3*	*ZIP3*	Hippocampus pyramidal neurons [62]	Seizure-induced CA1degeneration [62]	Unknown
*SLC39A4*	*ZIP4*	Choroid plexus [15]	Glioma [63]	Unknown
*SLC39A6*	*ZIP6*	Hippocampal pyramidal apical membrane of choroid plexus [17]	Unknown	Unknown
*SLC39A9*	*ZIP9*	Trans-Golgi network [64]	Unknown	Unknown
*SLC29A13*	*ZIP13*	Golgi apparatus [14]	SCD-EDS [65]	Unknown

### ZnT subfamily members in the brain

ZnT1 was the first member identified by isolation from a rat kidney cDNA expression library [14]. It localizes to the plasma membrane and is ubiquitously expressed [66]. In the brains of mice, ZnT1 is highly expressed in dendritic spines and synaptic membranes [12]. Besides, ZnT1 interacts with GluN2A-containing NMDA receptors, forming GluN2A/ZnT1 complexes [67]. Thus, in addition to exporting zinc to extracellular space, ZnT1 plays an important role in the modulation of postsynaptic receptors.

ZnT3 and ZnT4 are both expressed in the brain, but in different regions. Acting as a regulator of cognition and learning, ZnT3 was cloned by screening of a mouse λ library based on its homology to the ZnT2 transporter [14]. ZnT3 localizes to the synaptic vesicular membrane and is abundant in the hippocampus and cortex. In the brains of ZnT3 knockout mice, zinc is nearly undetectable in synaptic vesicles, suggesting that ZnT3 is responsible for transporting zinc into vesicles [54, 68]. Furthermore, ZnT3 also exists in the choroid plexus epithelial cells, the Bergman glial cells of mouse cerebellar cortex and the postganglionic neurons of mouse superior cervical ganglion [13]. However, ZnT3 may exert some undiscovered functions in the brain. ZnT4 was initially identified as the vital gene involved in the lethal milk mutant [69], and was later detected abundantly in mammary brain and glands, especially in the lysosomal and endosomal compartments, but with low expression in other tissues [53]. Perhaps, ZnT4 mainly facilitates the entry of zinc into secretory vesicles.

ZnT5 has homology with ZRC1 in yeast, and is widely expressed in all human tissues. Interestingly, ZnT5 and ZnT6 are both restricted to the membrane of the Golgi apparatus in mammalian cells, including neurons. ZnT5 and ZnT6 form hetero-oligomeric complexes for transporting zinc to tissue-nonspecific alkaline phosphatase (TNAP) [48]. Another ZnT transporter, ZnT7, also exists in the membrane of the Golgi apparatus. ZnT7 has homology with ZnT1, which is ubiquitously expressed in mouse tissues, particularly in the liver and small intestine. It is also found in the brain, kidneys and lungs [58]. The precise roles of ZnT5, ZnT6 and ZnT7 are obscure. Perhaps ZnT7 interacts with ZnT5 and ZnT6 to transport zinc to the secretory pathway and controls zinc homeostasis in the neuron.

Currently, there is limited knowledge regarding ZnT10. ZnT10 is highly homologous to ZnT1 and is mainly expressed in the fetal brain and fetal liver tissues. Hence, it can be speculated that ZnT10 is a crucial factor in fetal development [14]. ZnT10 is associated with PD and AD [49, 60], but whether ZnT10 transports zinc or manganese is controversial. Under normal conditions of cultured neuroblastoma cell lines, ZnT10 localizes at the Golgi apparatus. However, if the extracellular zinc concentration is higher than 100 μM, ZnT10 translocates to the plasma membrane and its mRNA expression level decreases [70]. Interestingly, other researchers have found that ZnT10 may primarily transport manganese rather than zinc [49, 71, 72]. Structurally, ZnT10 lacks a His-rich region that is similar to the manganese transporter shCDF8 in *Stylosanthes hamate* [73], whereas mutations in ZnT10 cause manganese disturbance [49].

### ZIP subfamily members in the brain

There are fewer studies on ZIPs relative to ZnTs. Human ZIP1 was identified through its homology with the *Arabidopsis* ZIP1 transporter, and is ubiquitously expressed in human tissues [74, 75]. Belloni-Olivi et al. [15] found ZIP1 to be abundant in some areas of the brain in rats, including the hippocampus and the thalamus. It was found that ZIP1 localizes to the membrane of microglia, which is responsible for zinc uptake in astrocyte and microglial cells [16]. Microglia are resident immune cells in the brain. Researchers have shown that zinc is a key factor in transient global ischemia-induced microglial activation through sequential activation of NADPH oxidase and Poly (ADP-ribose) polymerase-1 (PARP-1). Interestingly, ZIP1 is involved in this process [76].

It was also found that ZIP3 and ZIP4 are expressed in the brain. ZIP3 is highly expressed in the testes and mammary cells. Qian et al. [76] discovered that ZIP3 was abundant in hippocampus pyramidal neurons, and knockout of ZIP3 can attenuate seizure-induced CA1 degeneration. ZIP4 is widely expressed in human tissues, such as the small intestine, stomach and kidneys, and it plays a fundamental role in the absorption of zinc in the small intestine. In rats, researchers found ZIP4 to be expressed in the choroid plexus [15]. ZIP4 is considered a marker of glioma, due to its higher expression in higher grade of gliomas with shorter overall survival [63].

Functioning as a zinc transporter, ZIP6 is widely expressed, and is localized to the plasma membrane. In particular ZIP6 is found in the hippocampal pyramidal and the apical membrane of the choroid plexus. The researchers found that the mRNA level of ZIP6 increased in the brain of neonatal rats when zinc was deficient [17].

There are a few studies describing ZIP9 and ZIP13 in the brain. ZIP9 was found to regulate zinc homeostasis in the secretory pathway [64], and was characterized as a membrane androgen receptor (mAR) [77]. ZIP13 plays a key role in the development of bone, teeth and connective tissue, and its defect is responsible for spondylocheirodysplastic Ehlers-Danlos syndrome (SCD-EDS) [65]. Jeong et al. [42] reported that SCD-EDS was caused by zinc deficiency in the endoplasmic reticulum (ER) rather than an overload, because the evidence showed that ZIP13 functioned to release labile zinc from vesicular stores for use in the ER and other compartments. However, Xiao et al. [71] found that ZIP13 mediated iron export in *Drosophila*, suggesting that SCD-EDS may also be caused by the failure of the iron delivery to the secretory compartments [78]. A very recent study found that the expression of ZIP9 and ZIP13 increased significantly with aging in the normal human brain [52].

## Altered expression of zinc transporters in AD brains

**Fig. 2:**
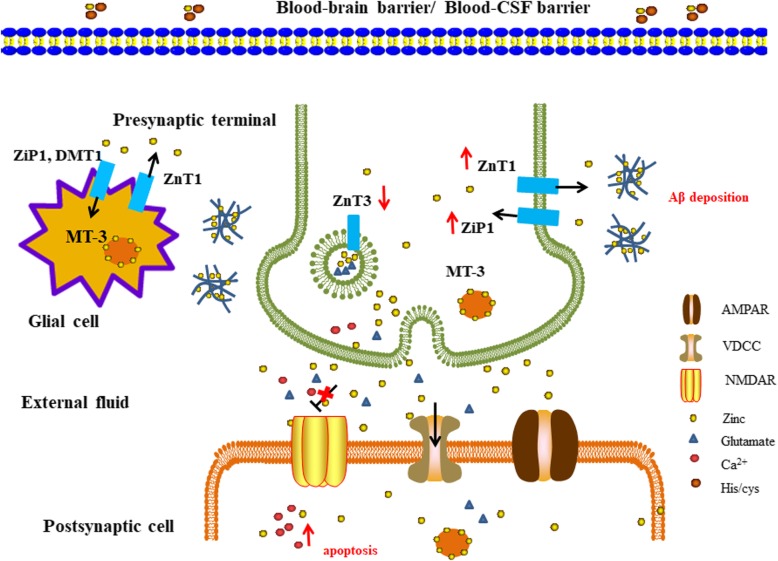
Schematic depiction of the major altered expression of zinc transporters in the neurons of developing AD. Exchangeable zinc ions across the blood-brain barrier/blood-CSF barrier by binding with His or Cys to form Zn (His)^2^ or Zn (Cys)(His)^−^, and then the complex is transferred into or out of the glial cells and neurons through zinc-binding proteins (ZIPs, ZnTs and DMT1). However, in AD patients, the expression levels of some major zinc transporters are altered, and this exacerbates Aβ deposition and toxicity. As shown in the figure, the highly upregulated ZnT1 pumps more zinc from presynaptic neurons and glial cells, which aggravates the deposition of Aβ proteins, and with the lower available zinc ions in the neuronal cytoplasm, upregulation of ZIP1 expression is induced to import zinc from the extracellular milieu to sustain the normal zinc homeostasis. However, this leads to a vicious cycle. In addition, the decreased expression level of ZnT3 leads to insufficient release of zinc into the cleft, and thus the inhibitory function of zinc on NMDAR will be impaired; as a result, more Ca^2+^ enters the postsynaptic cells, leading to apoptosis and cognitive disorders

There is increasing evidence that zinc transporters are connected with the formation of senile plaques in AD. Abundant expression and altered distribution of zinc transporters have been found in the senile plaques of APP/PS1 mice and human AD brains [56, 59]. ZnT1 and ZnT4 were present in the entire body of the senile plaques, ZnT3, ZnT5 and ZnT6 existed in the peripheral parts of the plaque, while ZnT7 was expressed in the center of the senile plaques [59]. Strikingly, the expression level of zinc transporters also changed abnormally during the progression of AD, including six ZnT transporters and one ZIP transporter (Table 1). Figure 2 shows a schematic depiction of the major altered expression of zinc transporters in the neuronal development of AD.

Generally, AD can be classified into four stages: preclinical AD (PCAD), mild cognitive impairment (MCI), early-stage AD (EAD) and late-stage AD (LAD) [79, 80]. MCI subjects have memory loss and a proven AD pathology but with normal daily activities, which usually fall between persons with little or no pathology and persons with AD, while PCAD subjects have evidence of AD neuropathology at autopsy but with normal cognitive functions [2, 80]. The T-tau or P-tau /Aβ42 ratio can be used to predict the progression of MCI to AD with high specificity [81]. The researchers showed that in PCAD patients, the expression level of ZnT1 in the hippocampus was low, accompanied by the increased zinc concentration in the cytoplasm [53]. Whereas, in patients with EAD and LAD, ZnT1 is elevated in the hippocampus gyrus [20]. Meanwhile, other researchers have also substantiated the elevated ZnT1 level in the brain of APP/PS1 mice [59]. Not only ZnT1, but also other ZnT proteins, such as ZnT4 and ZnT6, are additionally implicated in AD. ZnT4 is detected in the lysosomal and endosteum compartments in the hippocampal, and functions by loading cytoplasmic zinc in secretory vesicles [53]. ZnT6 is located in the membrane of the Golgi apparatus, where zinc binds to APP and inhibits the cleavage of APP at the α-secretase site [7, 57]. In the brains of PCAD patients, the level of ZnT1 decreases, concomitant with the significant elevation of ZnT6. In patients with EAD and LAD, both ZnT4 and ZnT6 levels are increased [55]. It can be interpreted that during the progression of AD, the increased level of ZnT1 leads to a higher zinc concentration in the extracellular space, aggravating the deposition of Aβ. On the other hand, when the expression of ZnT6 is higher, more zinc is brought into the trans-Golgi network (TGN), exacerbating Aβ aggregation through inhibition of APP cleavage by α-secretase. However, whether the perturbation of the abundance of ZnT1 affects NMDA receptor function and synaptic plasticity, and how ZnT4 influences the pathogenesis of AD remains unclear.

Some researchers found that the cortical ZnT3 level reduced with aging in wild mice and healthy humans, especially for AD patients the reduction was exacerbated [19]. 6-month-old ZnT3 knockout mice showed defects in learning and memory [18]. Similarly, in the brains of AD patients, the level of ZnT3 is significantly reduced [19]. The researchers proposed that the altered expression level of ZnT3 was due to the disturbance of zinc in synaptic vesicles, with an inadequate release of zinc into the synaptic cleft. Therefore, the inhibitory function of zinc on postsynaptic receptors is impaired, leading to cognitive deficits in AD [18, 82].

In AD patients, the mRNA level of ZnT10 decreases in the frontal cortex, which is parallel to the case in APP/PS1 mice [60]. We can speculate that the dysfunction of ZnT10 contributes to Aβ deposition and senile plaque formation, but whether it is the result of zinc or manganese transported by altered ZnT10 or both remains unclear.

ZIP1 is upregulated in the aging of human frontal cortex [52]. There is evidence that ZIP1 is associated with AD. In the brains of AD patients, the level of ZIP1 is significantly increased with disease progression and Braak staging [61]. This corresponds to the results obtained from the AD model of flies. In *Drosophila*, there also exist ZIPs and ZnTs. dZIP1 in flies is an ortholog of human ZIP1, which is enriched in the gut and responsible for zinc absorption [83]. Moreover, dZIP1 is also expressed in the brains of flies, and its expression level also rises with aging in Aβ42 flies [84]. These findings suggest that in the progression of AD, zinc interacts more and more with the Aβ and tau proteins, resulting in reduced availability of zinc in the cytoplasm. In order to maintain normal zinc homeostasis, ZIP1 is highly upregulated to uptake zinc from the extracellular milieu.

### Altered expression of metallothioneins in AD brains

The main function of metallothioneins is to transport, storage and regulate the cellular essential metals, such as zinc, copper, and iron. MT-3 plays an important role in brain zinc and copper homeostasis, which is expressed mainly in the brain, where it is particularly abundant in zinc-enriched neurons (ZEN), astrocytes, cerebellar cortex, and the early degenerated hippocampus in AD [85, 86]; In contrast, brain MT-1 and MT-2 are mainly expressed in glia cells and mainly in the astrocytes [85]. Considerable work has shown that MT is also abnormally expressed in AD brains [86–88], although some conflicting results have been reported. By using a large number of AD cases, it was proved that MT-3 expression is reduced in AD cases at mRNA level and at the protein level in temporal cortex, similar to those for Zn with regional redistribution [87]. In AD mouse, it is also found that MT-3 protein level is reduced in whole brain extracts [89]. However, MT-1 and MT-2 were reported to be up-regulated in AD cases [90–92]. It is proposed that the reduced MT-3 contribute to the loss of its protective effects and/or repair functions, leading to an exacerbation of the pathogenic processes, and the up-regulation of MT-1 and MT-2 may be a host defense response to elevated free intracellular Zn^2+^ levels, reflecting the pathology and inflammatory signals, and that all these changes imply a close relationship between MT and AD.

## Novel therapeutic targets for AD

Clioquinol (CQ or PBT1) and the 8-hydroxy quinoline analog PBT2 are two well-known metal ionophores that were developed to treat AD. They could act as chelators to bind excess metals in the brain, such as copper and zinc, as well as metal-protein attenuating compounds (MPACs), thus redistributing the two cations from enriched to depleted brain regions, indicating benefits for ameliorating AD both in mice AD models and in some clinical trials [9–11, 37, 93]. However, due to its depletion or redistribution of metal elements throughout the organism, the systemic administration of chelators may cause harmful side effects and create safety problems. PBT1 has been reported to cause myelinopathies in the CNS of a transgenic AD mouse model and wild-type mice [94]. Because of doubts about positive clinical benefits for AD patients, and misgivings about the safety of the drug [95], as well as problems with contamination during the manufacturing process [37], the planned phase III clinical trial of PBT1 has been abandoned, and the compound has been withdrawn. The metal chaperone of PBT2, as an improved chelator, could rapidly decrease soluble interstitial brain Aβ in a matter of hours and ameliorate the cognitive performance of AD mice [11]. Although PBT2 appeared to be safer, and resulted in a reduced amount of soluble plaque in Phase II trials in Europe (EURO) and Australia (IMAGINE), cognitive outcomes were different and restricted to two measures of executive function [96, 97]. Larger trials are needed to demonstrate its cognitive efficacy [98].

**Fig. 3:**
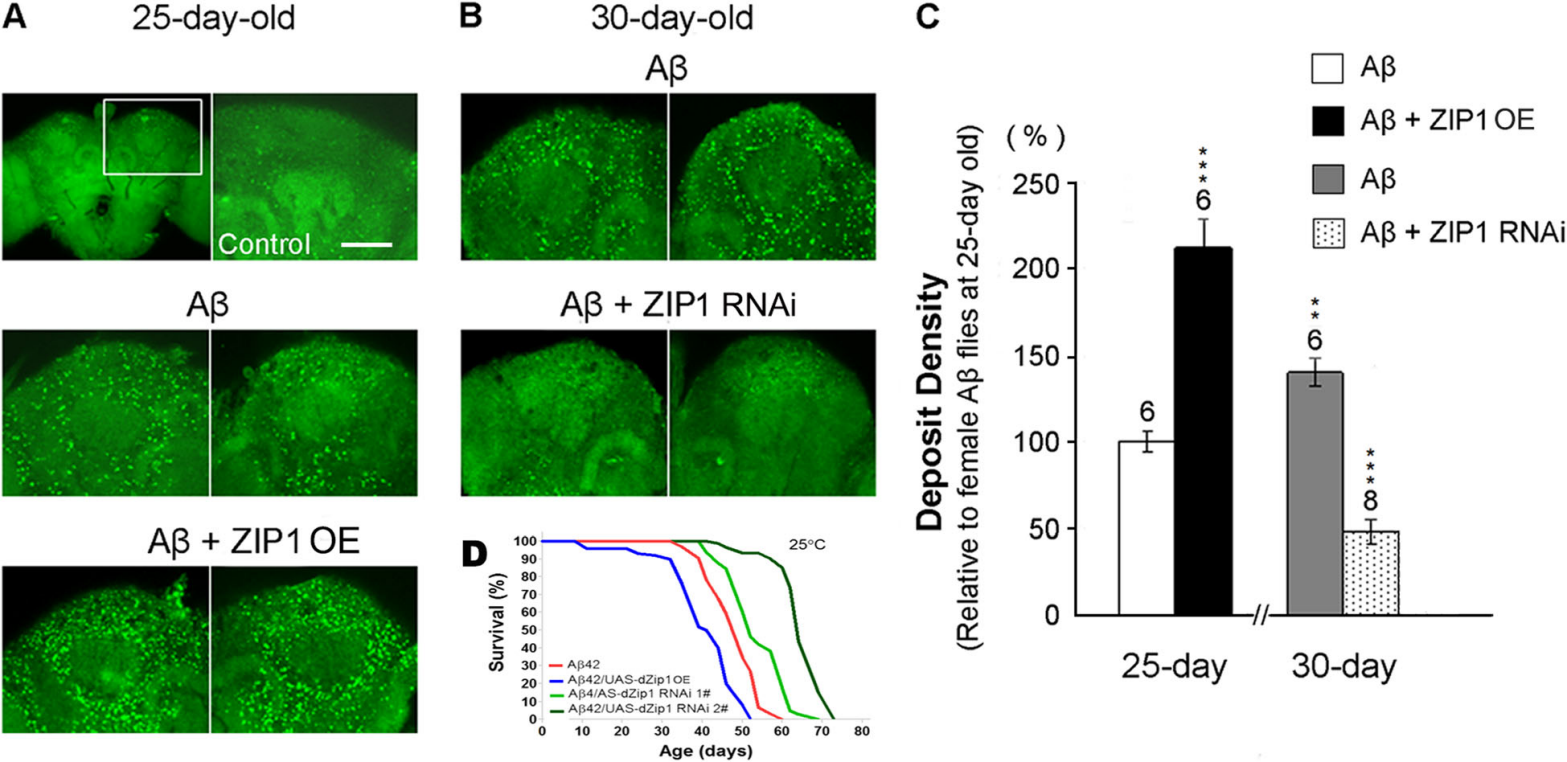
Modifying dZIP1 levels markedly influences the brain Aβ42 fibril deposition and lifespan in a *Drosophila* AD model. **a**-**b** Thioflavin-S (TS) staining was used to detect Aβ42 fibril deposits (bright green dots) in fly brains. Few deposits were found in the control brains (*Elav-Gal4*, top left) at 25 days after eclosion (dae). TS-positive deposits were found after Aβ42 expression in fly brains (*Elav-Gal4 > UAS-Aβ42*) at both **a** 25 and **b** 30 dae. **c** The quantitative content of Aβ42 deposits was summarized and expressed after normalization to 25-day old Aβ42 flies. The increase in Aβ42 deposits was age-dependent. Overexpression of dZIP1 in Aβ42-expressing brains (*Elav-Gal4 > UAS-Aβ42/UAS-dZIP1*) significantly increased fibril deposits at 25 dae, which was higher than 30 dae Aβ42 flies. However, inhibition of dZIP1 (*Elav-Gal4 > UAS-Aβ42/UAS-dZIP1 RNAi*) dramatically decreased deposit density at 30 dae, which was reduced compared to 25 dae Aβ42 flies. *t* test, **P < 0.01, ***P < 0.001. Data are expressed as means ± SEM. *n* = 6 or 8 hemispheres for each genotype. Scale bar: 25 μm. **d** dZip1 knockdown significantly prolongs the lifespan of Aβ42 flies. The percentage of survivorship was plotted against age (dae). Overexpression of dZIP1significantly shortened the lifespan of Aβ42 (elav-Gal4 > UAS-Aβ42) flies. Decreased dZIP1 levels (Elav-Gal4 > UAS-Aβ42/UAS-dZip1 RNAi) inhibited Aβ42 toxicity in a dose-dependent manner, and dZip1 RNAi #2 showed a more significant phenotype, in which dZip1-RNAi 2# (elav-Gal4 > UAS-Aβ42/UAS-dZip1-RNAi 2#) flies had much more reduced dZIP1 level than that of dZip1-RNAi 1# (elav-Gal4 > UAS-Aβ42/UAS-dZip1-RNAi 1#) flies. The differences shown are all statistically significant (*p* < 0.001). The reported *P* values are derived from Mantel-Cox log-rank statistical analysis

Since zinc transporters are critically involved in brain zinc homeostasis and AD pathological processes, it is possible to specifically modulate the expression level of brain zinc transporter to produce significant ameliorating effects on the progress of AD (as illustrated in Fig. 3). In the *Drosophila* AD model, Lang et al. [48] reported that *dZIP1* knockdown of Aβ42 flies led to a longer lifespan (Fig. 3d), a stronger climbing ability and fewer brain vacuoles than the control of Aβ42 flies, whereas *dZIP1* overexpression exacerbated the AD-like symptoms of Aβ42 flies. Furthermore, this study suggested an internal mechanism since the ameliorated AD-like symptoms were well correlated with significantly reduced Aβ42 fibril deposits (Fig. 3a-c) and its SDS-soluble forms, as well as reduced levels of zinc accumulation in brains of aged AD flies upon dZIP1 inhibition [84]. Recent cases reported by Huang et al. [99] also supports the hypothesis in another *Drosophila* AD model of tauopathy flies, which expresses a human Tau mutant (hTauR406W, or Tau*). Either knocking down dZIP1 or overexpressing dZnT1 significantly reduced the number of brain vacuoles and prolonged the lifespan of Tau* flies, demonstrating the ameliorating effects of zinc limitation through the genetic modulation of brain zinc transporters, where Tau phosphorylation and the toxicity produced by zinc-Tau binding were markedly reduced [99]. Another study in mice showed that knockout of *ZIP1* and *ZIP3* can attenuate seizure-induced neurodegeneration in hippocampal CA1 pyramidal neurons [62]. Moreover, 6 month-old ZnT3 knockout mice showed deficits in learning and memory [18, 68], and a further study showed that ZnT3 levels were negatively correlated with soluble Aβ [100], while the ablation of ZnT3 markedly reduced the plaque burden in *hAPP*^*+*^:*ZnT3*^−/−^ mice [101]. Roy et al. (2017) reported the design of compounds aimed at preventing MT-3 degradation in AD cases, and showed that Benzothiazolone-2 compounds significantly enhanced MT-3 protein and mRNA levels, suggesting its use as a lead compound for future drug design in the treatment of AD [102]. The surprising efficiency of the influence on AD-like symptoms in animal models, when modulating zinc transporters, suggests a novel strategy for the treatment of AD. In fact, correcting zinc homeostasis in the brains of AD patients by altering the expression level or traffic capacity of zinc transporters holds promise as an effective therapeutic strategy that should be further explored.

## Future questions and perspectives

**Fig. 4:**
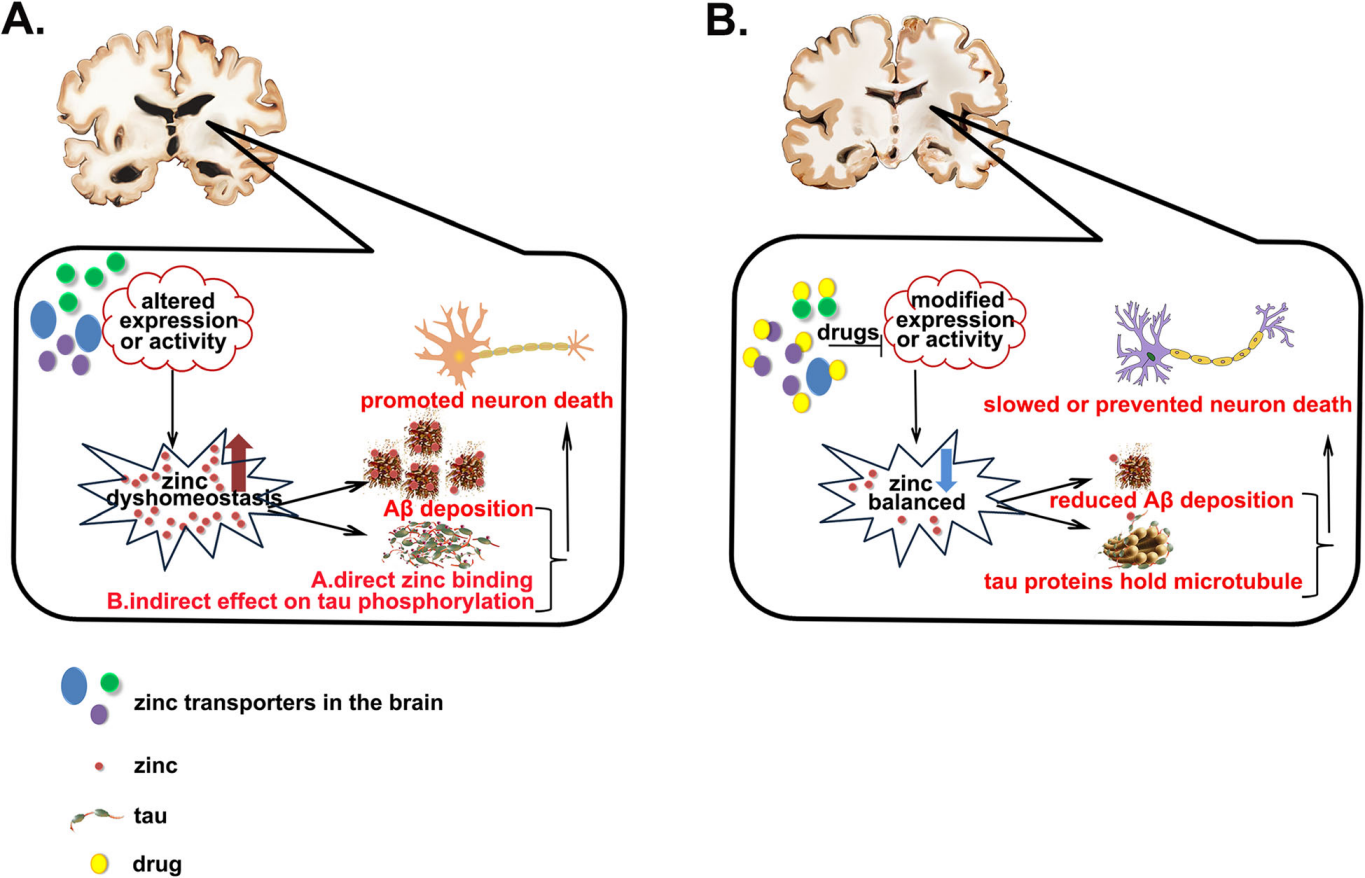
A proposed model illustrating the mechanism of AD progression and amelioration. The expression or alteration in activity of brain zinc transporters induce zinc dyshomeostasis, which aggravate Aß deposition, tau phosphorylation and tau-zinc binding exacerbated toxicity, increasingly promoting neuronal loss (**a**), while the leading compounds or drugs designed to specifically modify the expression or activity of brain zinc transport proteins have the potential to correct the disturbed zinc metabolism niche, thus leading to reduced Aß deposition, tau dissociation from microtubules and tau toxicity, which ultimately slows or prevents neuronal death in the onset and progression of AD (**b**)

In this review, we highlight the significant role of zinc transporters in the pathology of AD and catalogue their multiple pathological changes in disease progression. Figure 4 is a proposed model that illustrates the mechanisms of AD progression and amelioration strategy; however, some questions remain to be resolved. How do zinc transporters deliver zinc across the blood-brain barrier? Why do the expression levels of ZnT1, ZnT3, ZnT4, ZnT6, ZnT10 and Zip1 alter at different stages of AD? Furthermore, the way ZnTs and ZIPs exist in the glia to modulate zinc homeostasis in AD are also critical issues that must be resolved. We need to use AD models, such as *Drosophila*, to uncover how ZnTs and ZIPs interact to influence the development of AD pathology, and utilize the methods of gene therapy or the development of lead compounds to alter the expression or activity of zinc transporters in mice or higher animal models to investigate whether AD-like symptoms can be prevented or cured.

If such experiments are successful, in subsequent clinical trials, we need to find appropriate delivery methods for human-specific brain regions to specifically target the transporter mRNAs or proteins responsible for enhancing or weakening zinc levels or activity, in order to correct the disturbing zinc metabolism niche. Importantly, these genetic methods or novel pharmacological molecules used to reform the activity of zinc transporters may allow the discovery of novel therapies not only for AD, but also for other neurodegenerative diseases such as PD and HD. In summary, further exploration of the potential of transporter therapeutic targets may shed new light on the prevention or treatment of chronic neurodegenerative diseases.

## Data Availability

Not applicable.

## Abbreviations

AD: Alzheimer’s disease
AE: Acrodermatitis enteropathica
AMPA: α-amino-3-hydroxyl-5-methyl-4-isoxazole-propionate
APP: Amyloid precursor protein
Aβ: Amyloid β
BDNF: Brain-derived neurotrophic factor
CNS: Central nervous system
CQ: Clioquinol
EAD: Early-stage AD
ER: Endoplasmic reticulum
HD: Huntington’s disease
His: Histidine
LAD: Late-stage AD
mAR: membrane androgen receptor
MPP+: 1-Methyl-4-phenylpyridinium.
MTS: Metallothioneins
NFTs: Neurofibrillary tangles
NMDA: N-methyl-D-aspartate
PBT2: 5,7-Dichloro-2-[(dimethylamino)methyl]quinolin-8-ol
PCAD: Preclinical AD
PD: Parkinson’s disease
PS1: Presenilin 1
SCD-EDS: Ehlers–Danlos syndrome
SLC30: Solute carrier 30
SLC39: Solute carrier 39
TGN: Trans-Golgi network
TMDs: Transmembrane domains
TNAP: Tissue-nonspecific alkaline phosphatase
TrkB: Tropomyosin-related kinase B
VDCC: Voltage-dependent calcium channels
ZIP: Zrt- and Irt-like protein
ZnT: Zinc transporter
